# Diffuse pulmonary meningotheliomatosis – a case report

**DOI:** 10.7196/AJTCCM.2020.v26i1.012

**Published:** 2020-03-19

**Authors:** S D Maasdorp, J-M Nel, M Prins

**Affiliations:** 1 Pulmonology Division, Department of Internal Medicine, Faculty of Health Sciences, University of the Free State, Bloemfontein, South Africa; 2 Private Practice, Bloemfontein, South Africa

**Keywords:** pulmonary nodules, miliary, meningotheliomatosis, meningothelial-like

## Abstract

Diffuse pulmonary meningotheliomatosis is a rare condition of the lung that presents with nonspecific respiratory symptoms, and usually follows a benign course. It should, however, be considered in the differential diagnosis of a miliary pattern on chest-imaging studies, as illustrated in the case reported

## Case

**Fig. 1:**
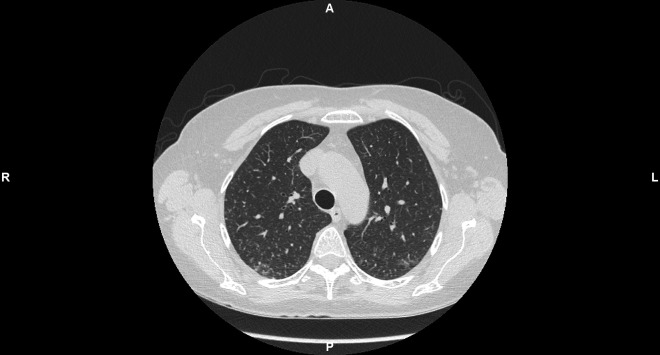
High-resolution computed tomography chest image showing numerous small pulmonary nodules in the lung apices bilaterally

We describe a case of a 58-year-old female who presented with complaints of dyspnoea on exertion and persistent non-productive coughing for 3 months. She also complained of dyspepsia and nausea. Her history was otherwise significant only for being on treatment with lamotrigine and donepezil after a diagnosis of frontal lobe syndrome secondary to previous traumatic brain injury. She was a non-smoker and did not have any occupational or organic dust exposure that could predispose her to pneumoconiosis or hypersensitivity pneumonitis. Previous operations included Nissen fundoplication. Her family history was not significant. On clinical examination, she looked well. Examination of the chest was unrevealing, and normal vesicular breath sounds were audible bilaterally. Chest X-ray and chest computed tomography (CT) images, however, revealed diffuse micronodular infiltrates in a miliary pattern bilaterally (Fig. 1). Lung function evaluation revealed normal spirometry, with an FVC of 3.03 L (117% of predicted), FEV_1_ of 2.56 L (117% of predicted) and FEV_1_ /FVC of 0.84, with no reversibility post bronchodilator. A gastroscopy performed as part of the diagnostic investigations for persistent cough found her to have reflux oesophagitis. Screening for autoimmune diseases was negative.

**Fig. 2:**
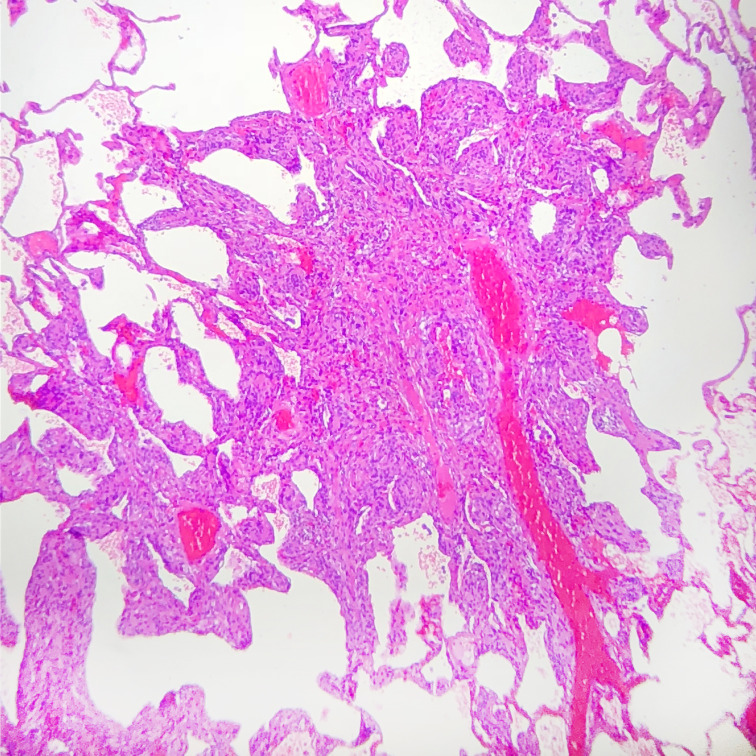
Haematoxylin and eosin stain of lung biopsy specimen at medium magnification revealing stellate-shaped meningothelial-like nodule.

Thoracoscopic lung biopsy was performed, with histology revealing pulmonary parenchyma with preserved lung architecture. Numerous small stellate-shaped nodular lesions were distributed throughout the lung tissue (Fig. 2). On higher magnification, the nodules comprised islands of epithelioid cells, with the appearance of arachnoidal cells. The cells showed eosinophilic cytoplasm, accompanied by bland, uniform and ovoid nuclei, some featuring pseudoinclusions. The immunohistochemical profile found the nodules to be positive for epithelial membrane antigen (EMA) and progesterone receptor (PR), confirming a histological diagnosis of meningothelial-like nodules. In combination with the clinical and radiological features, a final diagnosis of diffuse pulmonary meningotheliomatosis was made. Informed consent for publication was obtained from the patient, and ethics approval was provided by the Health Sciences Research Ethics Committee of the University of the Free State (ref. no. UFS-HSD2019/0226/2603).

## Discussion

Korn *et al*. ^[1]^ originally described these nodules as tumours resembling chemodectomas, based on morphological features. Later, immunohistochemical staining that was positive for CD56, PR, EMA and vimentin supported the meningeal rather than neuroendocrine derivation of these cells.^[2]^ In one of the largest surgical lung specimen biopsy series, pulmonary meningothelial-like nodules were found to have an incidence of up to 13.8%.^[2]^ They were found more commonly in female patients (female:male ratio of 2.2:1), mainly in patients >40 years old, and were more frequently noted in patients with underlying chronic lung disease.^[2]^ Although meningothelial-like nodules are usually single lesions, in rare cases they can be so numerous as to resemble a miliary pattern on chest-imaging studies, and the condition is then called diffuse pulmonary meningotheliomatosis.^[3]^ CT chest images usually reveals multiple small random nodules (0.1 - 3.0 mm) diffusely present in both lungs.^[4]^ Although diffuse pulmonary meningotheliomatosis usually has a benign course, it can be associated with other conditions such as pulmonary emboli, vasculitis, respiratory bronchiolitis-associated interstitial lung disease and lung cancer.^[5]^ No specific treatment is indicated for this condition. The patient was seen again in routine follow-up 6 months after her initial presentation. Her clinical condition, chest-imaging findings and lung functions remained unchanged.

## Conclusion

This case illustrates that diffuse pulmonary meningotheliomatosis should be considered in the differential diagnosis of an undefined ‘miliary-type’ pattern of pulmonary nodules on chest imaging.
